# Aryl Hydrocarbon Receptor Involvement in the Sodium-Dependent Glutamate/Aspartate Transporter Regulation in Cerebellar Bergmann Glia Cells

**DOI:** 10.1021/acschemneuro.4c00046

**Published:** 2024-03-08

**Authors:** Janisse Silva-Parra, Leticia Ramírez-Martínez, Cecilia Palafox-Gómez, Cristina Sandu, Esther López-Bayghen, Libia Vega, Guillermo Elizondo, Jaqueline Loaeza-Loaeza, Daniel Hernández-Sotelo, Luisa C. Hernández-Kelly, Marie-Paule Felder-Schmittbuhl, Arturo Ortega

**Affiliations:** † Departamento de Toxicología, Centro de Investigación y de Estudios Avanzados del Instituto Politécnico Nacional, Apartado Postal 14-740, Ciudad de México 07360, Mexico; ‡ Centre National de la Recherche Scientifique, Université de Strasbourg, Institut des Neurosciences Cellulaires et Intégratives, Strasbourg 00000, France; § Departamento de Biología Celular, Centro de Investigación y de Estudios Avanzados del Instituto Politécnico Nacional, Apartado Postal 14-740, Ciudad de México 07360, Mexico; ∥ Facultad de Ciencias Químico-Biológicas, Universidad Autónoma de Guerrero, Chilpancingo 39070, Guerrero, Mexico

**Keywords:** glutamate transporters, aryl hydrocarbon receptor, Bergmann glia, transcriptional regulation, environmental clues

## Abstract

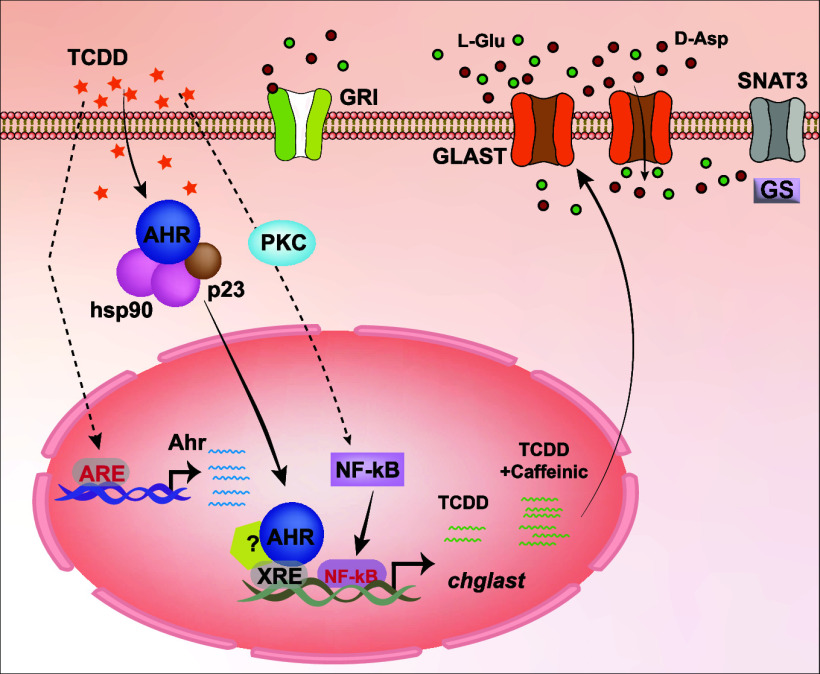

Glutamate, the major excitatory neurotransmitter in the vertebrate brain, exerts its functions through the activation of specific plasma membrane receptors and transporters. Overstimulation of glutamate receptors results in neuronal cell death through a process known as excitotoxicity. A family of sodium-dependent glutamate plasma membrane transporters is responsible for the removal of glutamate from the synaptic cleft, preventing an excitotoxic insult. Glial glutamate transporters carry out more than 90% of the brain glutamate uptake activity and are responsible for glutamate recycling through the GABA/Glutamate/Glutamine shuttle. The aryl hydrocarbon receptor is a ligand-dependent transcription factor that integrates environmental clues through its ability to heterodimerize with different transcription factors. Taking into consideration the fundamental role of glial glutamate transporters in glutamatergic synapses and that these transporters are regulated at the transcriptional, translational, and localization levels in an activity-dependent fashion, in this contribution, we explored the involvement of the aryl hydrocarbon receptor, as a model of environmental integrator, in the regulation of the glial sodium-dependent glutamate/aspartate transporter. Using the model of chick cerebellar Bergmann glia cells, we report herein that the aryl hydrocarbon receptors exert a time-dependent decrease in the transporter mRNA levels and a diminution of its uptake activity. The nuclear factor kappa light chain enhancer of the activated B cell signaling pathway is involved in this regulation. Our results favor the notion of an environmentally dependent regulation of glutamate removal in glial cells and therefore strengthen the notion of the involvement of glial cells in xenobiotic neurotoxic effects.

## Introduction

Glutamate (Glu) is the major excitatory neurotransmitter in the vertebrate brain; it exerts its actions through the activation of specific plasma membrane receptors and transporters present in neurons and glial cells. Glu-gated ion channels, known as ionotropic Glu receptors (GRI), are responsible for fast excitatory neurotransmission, whereas metabotropic Glu receptors (GRM) mediate slower excitatory signals.^1^ Both subtypes of receptors have been characterized in neurons and in glial cells as well as in other tissues besides the Central Nervous System (CNS).^2^ A membrane-to-nuclei Glu signaling is involved in transcriptional gene expression regulation, whereas a Glu-dependent increase in cytoplasmic Ca^2+^ participates in translational control.^3,4^ Ionotropic receptors are grouped in terms of their specific agonists in *N*-methyl-d-aspartate (NMDA), α-amino-3-hydroxy-5-methyl-4-isoxazolepropoionic acid (AMPA), and Kainate (KA) receptors. Several neurodegenerative diseases have common drivers, one of them is excitotoxicity, which is a consequence of the overstimulation of Glu receptors and results in neuronal and oligodendrocyte death.^5,6^

Excitatory Amino Acid Transporters (EAATs) are a family of sodium-dependent Glu transporters expressed in neurons and glial cells, responsible for the efficient removal of this amino acid from the synaptic cleft and thus preventing an excitotoxic insult. This gene family is composed of five members (EAAT1–5), of which the sodium-dependent Glu/aspartate transporter (EAAT1/GLAST) and the Glu transporter 1 (EAAT2/GLT-1) were described as glial specific, although GLT-1 has also been detected, albeit at low concentrations, in hippocampal synaptic terminals.^7,8^ Glial Glu transporters are known to carry out more than 90% of the brain Glu uptake activity, and its recycling depends on a neuronal/glial biochemical coupling known as the Glu/Glutamine shuttle.^9^ Within the glial compartment, Glu is rapidly metabolized to glutamine by the glial enriched enzyme glutamine synthetase (GS) to be released in a Na^+^-dependent manner through the neutral amino acid transporters of the N family, mainly the sodium-dependent neutral amino acid transporter 3 (SNAT3). Glutamine is taken up by the presynaptic terminals *via* another SNAT member, in this case, SNAT2. Glutamine is deaminated in the presynaptic terminal and the produced glutamate is packed into synaptic vesicles *via* vesicular glutamate transporters (VGLUTs) completing the neurotransmitter turnover. Although this shuttle has been questioned, its existence has been documented in several brain areas such as the cerebellum and retina.^10^ In fact, GS inhibition disrupts glutamatergic transmission.^11^ A tight regulation of glial Glu transporters is needed for smooth glutamatergic transmission. EAATs are regulated at the transcriptional, translational, and plasma membrane levels (reviewed in a previous study^7^). Within the cerebellum, GLAST/EAAT1 is the prominent transporter and is localized in Bergmann glia cells (BGC) which surround the most abundant glutamatergic synapses in the central nervous system (CNS), those established between the axons of the granule cells (parallel fibers) and the Purkinje cells.^12^ Using chick cerebellar BGC cultures, several aspects of GLAST/EAAT1 regulation have been reported (reviewed in refs (9 and 13)), including its transcriptional control, in fact, the promoter region of the chick GLAST/EAAT1 gene (*chglast*) has been analyzed.

Within the glial compartment, several sensors for multiple endogenous, metabolic, microbial, and environmental ligands are present. One of these sensors is the ligand-activated transcription factor aryl hydrocarbon receptor (AhR). This heterodimeric transcription factor belongs to the basic-helix–loop–helix (bHLH) family and is a member of the gene superfamily that harbors the Per-Arnt-Sim (PAS) domain.^14,15^ The PAS domain can bind and sense endogenous or xenobiotic small molecules, such as molecular oxygen, cellular metabolites, or polyaromatic hydrocarbons. AhR target genes contain a specific DNA binding sequence (5′-TNGCGTG-3′) known as the *xenobiotic response element* (XRE).^16^ The inactive AhR is localized in the cytoplasm, once it binds its agonist, it is activated and translocated into the nucleus, where it binds the XRE sequence present in its target genes, or forms heterodimers with other heteromeric transcription factors, such as nuclear factor kappa light chain enhancer of the activated B cell (NF-kB)^17^ or the clock gene, Brain and muscle arnt-like (BMAL1).^18^ The interaction between AhR and the NF-kB/Rel family increases the transcriptional landscape of the target genes regulated by this receptor. When the RelA/AhR complex is formed, their canonic target genes are avoided, resulting in an apparent negative regulation, and the heterodimer regulates genes such as c-Myc. The RelB/AhR complex binds to the XRE as well as to the NF-kappaB binding site regulating target genes of the AhR and NF-kappaB signaling pathway.^17^

The AhR can be activated by endogenous and exogenous molecules such as environmental pollutants, e.g., the dioxin 2,3,7,8-tetracloro-p-dioxin (TCDD).^19,20^ The neurotoxic effects of TCDD in the cerebellar granule cells were originally described by Kim and Yang.^21^ Moreover, TCDD exposure has been linked to abnormal cerebellar maturation,^22^ processes in which BGC are critically involved, through the Ying Yang 1 (YY-1) transcription factor.^23^ Interestingly enough, YY-1 downregulates GLAST/EAAT1 expression in BGC,^24^ opening the possibility of the involvement of cerebellar radial glia cells in TCDD cerebellar neurotoxicity. In this context, in the present contribution, we use chick cerebellar cultured BGC, to explore a plausible role of AhR in GLAST/EAAT-1 gene expression regulation and thus in TCDD cerebellar neurotoxicity. Besides canonical and well-described transcription binding sites in the *chglast* promoter such as Sp1, AP-1, NF-kB, NFAT, N-myc, CREB, and YY1,^25^ we identified an AhR consensus binding sequence. Our results describe a TCDD-dependent GLAST/EAAT1 downregulation favoring the notion of an excitotoxic insult triggered by this dioxin in BGC that might contribute to cerebellar neuronal death.

## Results

### AhR DNA-Binding Sequences Are Found within the *chglast* Promoter

**Figure 1:**
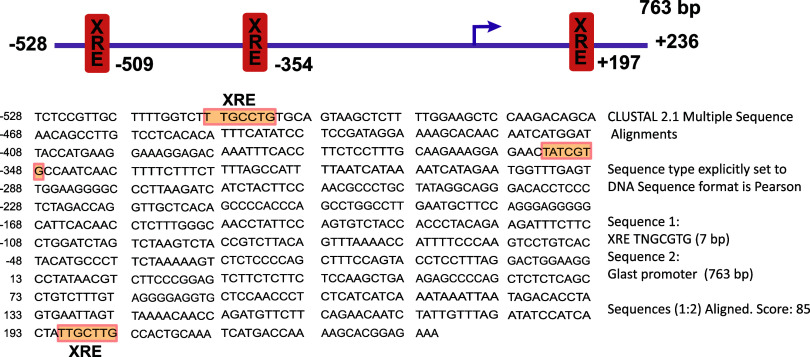
XRE sequences present on the *chglast* promoter. The *in silico* analysis shows three plausible XRE binding sites at −509, −354 and +197 positions of the *chglast* promoter.

The AhR receptor is a ligand-dependent transcription factor interacting with target genes through regulatory sequences known as XRE or DRE. To clarify the possible regulation of GLAST/EAAT1 by the AhR, we decided to look for XRE binding sites within the *chglas*t promoter previously reported by us,^26^ using a multiple sequence Alignments program.^16^ Three *bona fide* XRE sites were mapped at −509, −354, and +197 with an alignment score of 85. The *in silico* analysis is represented in Figure 1. These results prompted us to go directly to the GLAST/EAAT1 functional studies.

### TCDD Regulates [^3^H]-d-Aspartate Uptake

**Figure 2:**
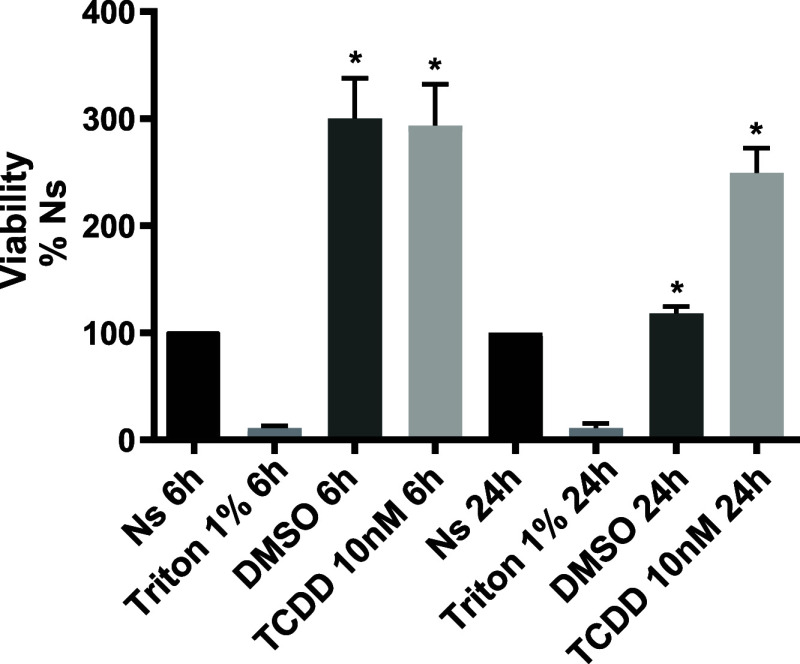
Effect of TCDD on the BGC viability. BGC Monolayers were treated with 10 nM of TCDD for 6 or 24 h. TCDD was diluted initially in DMSO and subsequently in culture medium. The DMSO concentration was 0.02%, that was also used alone as a Control. Ns: nonstimulated. Values represent mean ± SEM of three independent experiments. A one-way ANOVA was performed to determine whether there were significant differences between groups with a Dunnett test (Prism 9 software) *p < 0.05.

TCDD is the most toxic member of the dioxins group which are highly environmental pollutants.^27^ The initial toxic actions of this dioxin are exerted through its binding to the AhR and the subsequent activation of the established signaling cascade that results in *cyp1a1* transcription.^28^ Therefore, we chose to use TCDD to explore if AhR activation is involved in GLAST/EAAT1 activity regulation, the sole Glu transporter expressed in these cells.^29^ First, we decided to rule out any TCDD cytotoxic effect in our culture system. We exposed confluent BGC monolayers to a fixed 10 nM TCDD concentration for 6 and 24 h and measured cell viability with the MTT assay. An increase in the formazan production at 6 and 24 h was observed upon TCDD indicating an increase in the metabolic activity of the treated cells (Figure 2). Since the purpose of these experiments was to explore a plausible cytotoxic effect of TCDD in BGC, the fact that we did not get any reduction in the formazan production upon TCDD settles the point. Whether 24 h results in cell proliferation is out of the scope of this contribution which seeks to investigate a possible AhR-dependent regulation of glial Glu uptake. As expected, 0.02% DMSO, which was used as the TCDD solvent, has no effect on cell viability after 24 h, allowing us to use this vehicle for further experiments. Also is relevant to point out that exposure to 1% Triton X-100 diminished cell viability as expected.

**Figure 3:**
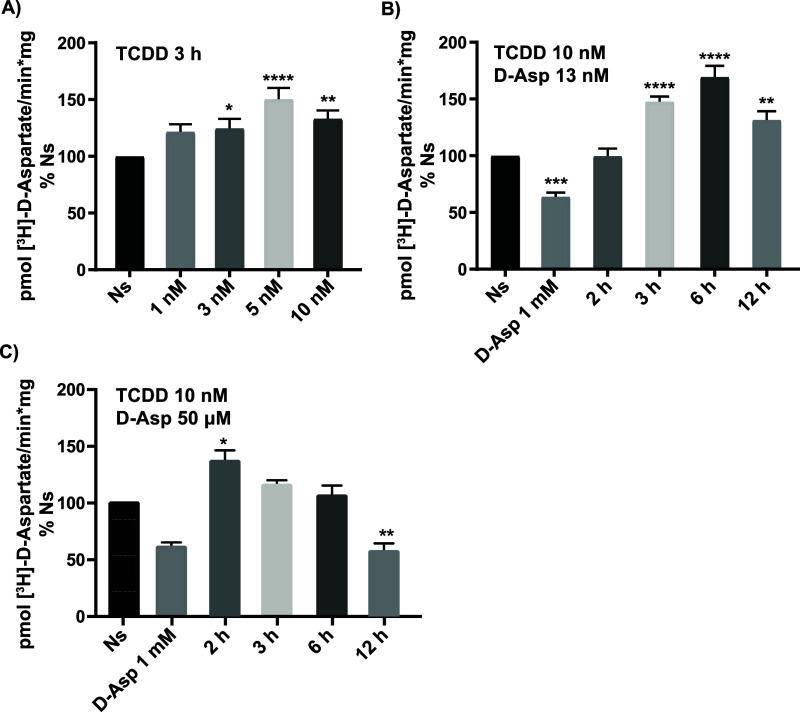
TCDD effect in [^3^H]-d-Aspartate Uptake in BGC. BGCs were exposed to different TCDD concentrations for 3 h (Panel A) and different time periods with 10 nM TCDD (Panel B). After the treatment, the uptake of [^3^H]-d-Aspartate (0.4 μCi/ml, for 30 min) was performed. In Panel C, confluent monolayers were exposed to 10 nM TCDD and the uptake assay was performed with a 50 μM d-Asp final concentration. TCDD was diluted initially in DMSO and subsequently in culture medium. The highest DMSO concentration was 0.02%. Ns: Non-stimulated. Data is the average of three independent experiments performed in quadruplicates. Values represent mean ± SEM. A one-way ANOVA was performed to determine whether there were significant differences between groups with a Dunnett test (Prism 9 software) *p < 0.05, **p < 0.01, ***p < 0.001, ****p < 0.0001.

Next, we evaluated the GLAST/EAAT1 activity in BGC confluent monolayers *via* a [^3^H]-d-Aspartate uptake assay. The results are shown in Figure 3, a dose (panel A) and time-dependent increase in [^3^H]-d-Aspartate uptake is present after the exposure to TCDD. It should be noted that these [^3^H]-d-Aspartate uptake assays were done at a very low single d-Asp concentration (13 nM) in order to improve the assay sensitivity and taking into consideration that BGC are enriched in glial Glu transporters as has been documented over the years.^7,12^ When the time dependence TCDD effect was determined at a higher d-Aspartate concentration (50 μM, which is in the range the transporter affinity^29^) a 12 h TCDD exposure results in a significant reduction in [^3^H]-d-Aspartate uptake. These results clearly demonstrate that AhR regulates the GLAST activity in a time- and dose-dependent fashion. Note that a 30 min preincubation with 1 mM d-Asp down regulates the transporter activity as previously reported.^7,12^

### TCDD Effect on [^3^H]-d-Aspartate is Sensitive to Actinomycin-D

**Figure 4:**
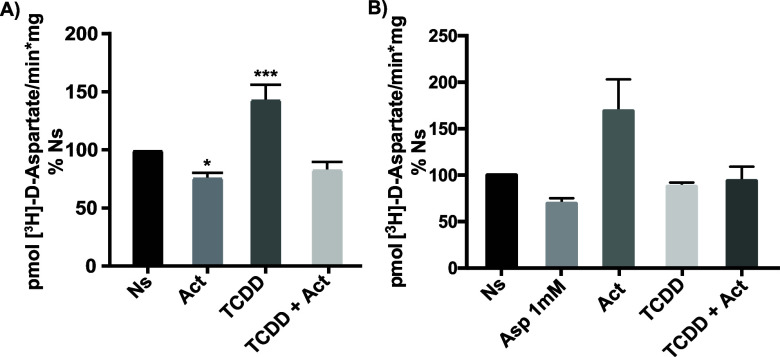
Actinomycin D pretreatment prevented the TCDD effect. (A) BGC were preincubated with 4 μM Actinomycin-D for 30 min, then treated with 10 nM TCDD for 3 h. The uptake was performed as in Figure 3 for panel A (13 nM d-Asp final concentration); for panel B, a 50 μM d-Asp final concentration was used. TCDD was diluted initially in DMSO and subsequently in a culture medium. The DMSO concentration was 0.02%. Three independent experiments, performed in quadruplicate, are shown. Values represent mean ± SEM. A one-way ANOVA was performed to determine whether there were significant differences between groups with a Dunnett test (Prism 6 software) *p < 0.05, ***p < 0.001.

To define a possible transcriptional role of AhR in the regulation of GLAST/EAAT1 uptake, we pretreated the cells with the RNA pol II inhibitor Actinomycin-D at a 4 μM concentration for 30 min and the added 10 nM TCDD for 3 h. The Actinomycin D treatment prevented the TCDD-mediated change in the GLAST/EAAT1 function. These results show that in the absence of RNA pol II activity by the pretreatment with Actinomycin D, TCDD does not modify the [^3^H]-d-Aspartate uptake (Figure 4) strongly suggesting that the AhR agonist effects are mediated by transcriptional events.

**Figure 5:**
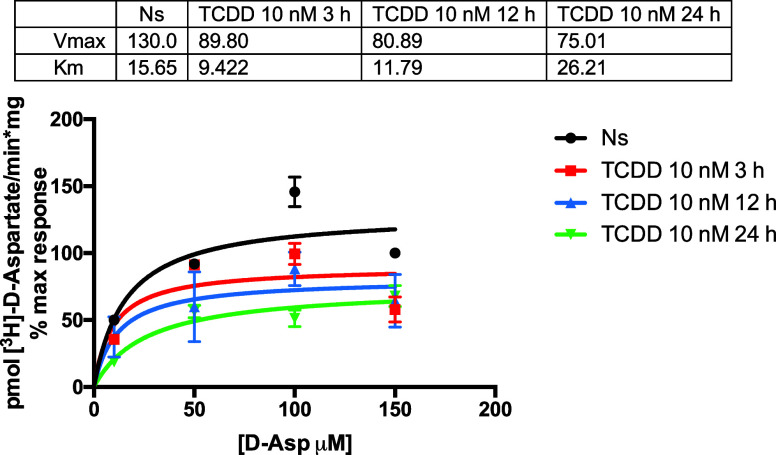
Michaelis–Menten analysis of the effect of TCDD exposure on GLAST/EAAT1 kinetic parameters. Confluent BGC monolayers were exposed to 10 nM TCDD for 3, 12, or 24 h. TCDD was diluted initially in DMSO and subsequently in a culture medium. The DMSO concentration was 0.02% The [^3^H]-d-Asp uptake was measured at different ligand concentrations (0–150 μM). Three independent experiments performed in quadruplicates were done and the kinetic parameters were determined after a nonlinear regression with the Prisma 6 software.

To characterize the TCDD effect, we performed a Michaelis–Menten analysis to establish the kinetic parameters of [^3^H]-d-Aspartate uptake in control and TCDD-treated cells for 3, 12, and 24 h. A sharp decrease in Vmax was obtained for all of the time periods tested (Figure 5). We could also detect small variations in the affinity of the transporter albeit not as significant as the decrease in Vmax, which could be interpreted as a diminution in plasma membrane transporters. TCDD exposure results in a significant decrease in the uptake activity that is sensitive to the RNA pol II inhibitor.

### TCDD Represses *chglast* Transcription *via* NF-κB

**Figure 6:**
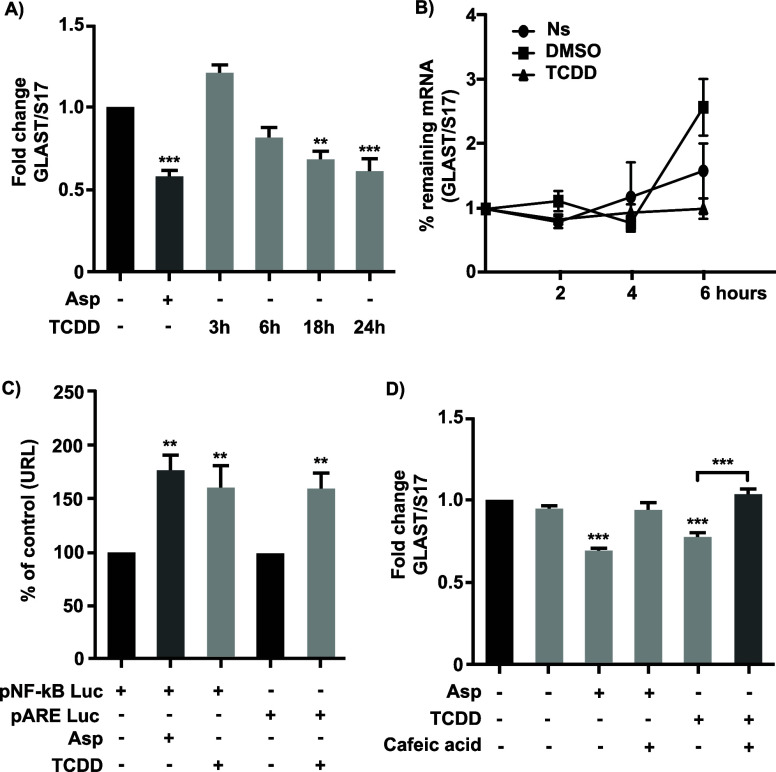
(A) BGC were treated with D-Asp (150 μM), TCDD (10 nM), or 0.02% DMSO (TCDD treated-like TCDD vehicle) for 3, 6, 18, and 24 h. TCDD was diluted initially in DMSO and subsequently in a culture medium. The DMSO concentration was 0.02%. Total mRNA was extracted, and *chglast* mRNA was quantified by RT-qPCR. Each bar is the mean ± SEM from three independent experiments by triplicate and normalized using the ribosomal protein S17 gene *S17*. (B) BGC were treated with D-Asp (150 μM), TCDD, or DMSO (TCDD treated like TCDD vehicle) for 24 h, *chglast* mRNA half-life was determined by stopping transcription with 4 μM Actinomycin A, and after 0, 2, 4, and 6 h, *chglast* mRNA levels were determined by RT-qPCR. (C) BGC were transfected with pNF-kB Luc or pARE Luc using Lipofectamine. Twenty-four h post-transfection, luciferase reporter assays measured NF-kB or ARE transcription factors’ activation. Data were normalized to control (DMSO), and each bar represents the mean ± SEM from 3 independent experiments performed in triplicates. (D) Confluent BGC cultures were pretreated with DMSO (control, TCDD vehicle 0.1%) or Caffeic Acid (8.8 μM), and treated with Asp (150 μM), TCDD for 24 h, total mRNA extraction was performed and *chglast* mRNA levels were measured by RTqPCR. Results the mean ± SEM from 3 independent experiments by triplicates and normalized using *S17.* One-way ANOVA was performed to determine whether there were significant differences between groups with a Bonferroni test (Prisma 6 software). *p < 0.05; **p < 0.01; ***p < 0.001.

At this stage, it was clear that the TCDD effect most possibly reflects repression of *chglast* transcription; to further support this interpretation, we decided to evaluate the mRNA levels after different time periods of a 10 nM TCDD exposure. The results are listed in Figure 6. A significant reduction in *chglast* mRNA levels is present after 18 h of TCDD exposure. As a control, BGC were exposed to 1 mM d-Asp, and the already characterized decrease in *chglast* mRNA levels was detected.^30^ To rule out a TCDD-mediated decrease in mRNA stability, we measured the half-life of this transcript by stopping transcription with 4 μM Actinomycin-D and measuring chglast mRNA levels at 0, 2, 4, and 6 h post-treatment, the results are depicted in panel B of Figure 6, *chglast* mRNA stability is not affected by TCDD exposure. Previous reports from our group indicate that NF-κB is a negative regulator of the *chglast* expression.^30^ Moreover, a selective complex between activated NF-κB and AhR capable to regulate gene expression has also been described.^17^ Therefore, we decided to test this latter possibility. To this end, we measured the transcriptional response in BGC transfected with two different constructs, the first one containing 5 NF-κB consensus binding sites in front of the luciferase reporter gene and the other construct bearing 5 ARE sequences. The transfected cells were treated with 1 mM d-Asp (positive control^30^) or 10 nM TCDD. The results are clear: TCDD exposure is capable of inducing the transcriptional activation of both reporter genes, favoring the notion of a TCDD-dependent *chglast* transcriptional repression through an NF-κB signaling cascade. This interpretation is further supported by the fact that caffeic acid (an NF-κB signaling cascade blocker^30^) is capable of preventing the TCDD effect (Figure 6, Panel D).

**Figure 7:**
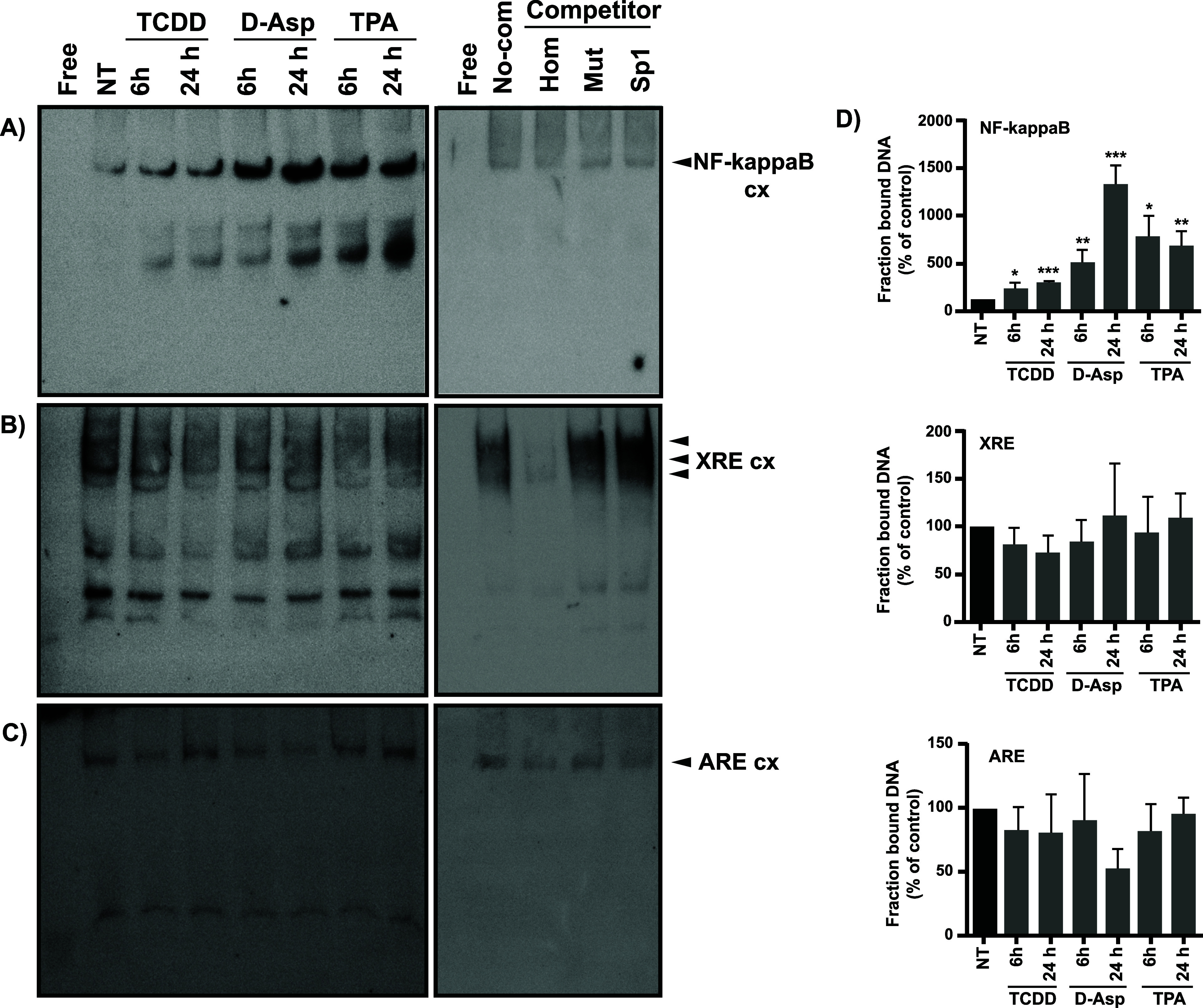
TCDD increases the NF-kB binding on its target sequence in the *chglast* promoter. EMSA was performed with nuclear extracts from BGC treated by 6 or 12 h with TCDD (10 nM), d-Asp (1 mM), or TPA (100 uM) as indicated in each panel. Nuclear extracts of BGC were incubated with (A) biotin-NF-kB probe and (B) biotin-XRE probe, both performed using the *chglast* promoter sequence, and (C) biotin-ARE probe that corresponds to the NRF2 target sequence on the AhR promote. In all the cases, in the middle panel, the competition assays with homologous probes (Hom), mutated consensus sequences (Mut), and heterologous probes (Sp1) are shown. For competition experiments, equal amounts of nuclear extracts were preincubated with unlabeled 10× cold probes for Hom, Mut, and Sp1. The expected decrease in labeled complexes was detected with the Hom sequence in all cases. (D) The DNA–protein complexes (cx) were quantified with Image J software and the bars represent the mean ± SEM from 3 independent experiments. Student′s *t* test was performed to determine significant differences between evaluated treatments (Prisma6 software). *p < 0.05; **p < 0.01; ***p < 0.001.

To support these findings, electrophoretic mobility shift (EMSA) assays were performed with nuclear extracts prepared from confluent BGC monolayers treated with TCDD (10 nM, 0.02% DMSO), 1 mM d-Asp, or 100 nM 12-O-Tetradecanoylphorbol-13-acetate (TPA) for the indicated time periods. The NF-κΒ probe corresponds to the NF-κΒ DNA binding site within the reported *chglast* promoter.^31^ The XRE and the ARE probes correspond to the reported binding sites in the *chglast* and AhR promoter, respectively.^32^ The sequences are shown in Table 1. The results are listed in Figure 7.

**Table 1:** Sequences of the EMSA Probes

transcription factor	probe sequence
NF-kapaB	Foward 5′-AGGCAGGGACACCTCCCTCTAG-3′
	Reverse 5′-CTAGAGGGAGGTGTCCCTGCCT-3′
ARE	Forward 5′-GGCGGCGGCGCTGTCAGGCC-3′
	Reverse 5′-GGCCTGACAGCGCCGCCGCC-3′
XRE	Forward 5′-GGAGAACTATCGTGCCAATC-3′
	Reverse 5′-GATTGGCACGATAGTTCTCC-3′
NF-kapaB mut	Forward 5′- AGGCAcccACACCTaatTCTAG-3′
	Reverse 5′- CTAGAattAGGTGTgggTGCCT-3′
ARE mut	Forward 5′ GGCGGCGtaGCTtgacGGCC-3′
	Reverse 5′ GGCCgtcaAGCtaCGCCGCC-3′
XRE mut	Forward 5′- GGAGAACgATacatCCAATC-3′
	Reverse 5′- GATTGGatgtATcGTTCTCC-3′

As depicted in Figure 7, an evident increase in NF-κΒ binding to the *chglast* promoter is present upon TCDD exposure, and as expected d-Asp and TPA also increase the binding,^30^ supporting our interpretation of suggesting that TCDD decreases GLAST/EAAT1 expression through *a negative transcriptional regulation of*NF-κΒ. Of relevance are the results presented in Panels B and C in which TCDD treatment does not have a clear effect on the XRE binding in the chglast promoter or ARE binding to the AhR promoter. As expected, the competition experiments with a 10× excess of the cold probe as well as a lack of competition with the mutated probes validate our findings (Figure 7, *right panel).*

## Discussion

As previously reported by our group, the transcriptional control, as well as the function of GLAST/EAAT1 depends on the substrate translocation process.^30^ The important role of this transporter goes beyond the clearing excess of Glu from the synaptic cleft after neuronal communication, its involvement in the recycling of the excitatory amino acid through the Glutamate/glutamine shuttle places it as a component in what now is known as tripartite synapses.^33^ The activity of GLAST/EAAT1 is regulated by a rapid process dependent on the amount of active membrane transporters and their affinity and in the long term through transcriptional control. It has been documented that the *glast* promoter activity is modulated positively by IGF-1 (Insulin-like growth factor-1), FGF (Fibroblast growth factor), EGF (Epidermal growth factor), TGF alpha, estrogen, HDACs inhibitors and negatively by TNF alpha, YY-1 and environmental toxins as manganese.^24,25,34−39^ Despite the key role of GLAST/EAAT1 in several neurological disorders, the molecular mechanisms involved in its transcriptional regulation are not completely understood. In this context, we decided to evaluate the effects of AhR, an environmental sensor receptor expressed in the *Gallus* central nervous system,^40^ since within the *chglast* promoter, *cis* elements capable of binding AhR are present (Figure 1).

The exposure of BGC monolayers to the AhR agonist TCDD modifies the [^3^H]-d-Aspartate uptake and a change in GLAST/EAAT1 activity is recorded after a 3 h exposure to a 3 nM TCDD concentration, an effect that is prevented by the RNA polymerase blocker (Actinomycin D), demonstrating the transcriptional nature of the dioxin effect. The complete characterization of the TCDD effect in terms of the enzymatic activity of GLAST/EAAT1 demonstrated that in fact, the dioxin diminishes the uptake activity, favoring the notion of a decrease in the amount of active membrane transporters in an RNA pol II-dependent manner. The apparent discrepancy in the TCDD effect (uptake increase, Figure 3 panels A and B, compared to Panel C) of Figure 3 and Figure 5 lies in the d-Asp final concentration used (13 nM or 50 μM) as annotated in each figure, except, of course, in the Michaelis–Menten analysis shown in Figure 5. The rationale for the use of the low (13 nM) concentration was to have a high specific activity and therefore sensitivity, given the fact of the abundance of GLAST/EAAT1 in BGC.^29,12^ The analysis of the Michaelis–Menten experiments demonstrated that TCDD exposure resulted in a decrease in GLAST/EAAT1 activity, so the apparent increase initially found was the result of the low d-Asp concentration used initially. In support of this interpretation, 12 h exposure to 10 nM TCDD results in a reduced [^3^H]-d-Aspartate uptake when the final d-Asp concentration is 50 μM a concentration well in the range of the reported GLAST/EAAT1 K_*M*_ (30 μM)^29^ (Figure 5).

In chicken, two isoforms of AhR have been reported: AhR1 and AhR2, the latter corresponds to ≅ 40% of the liver AhR, tissue in which this isoform is enriched. Most other tissues, including the brain, express mainly the AhR1. The chick version of AhR is highly homologous to that of mice and the human versions of the receptor.^41^ As pointed out earlier, *in silico* analysis of the *chglast* promoter showed the presence of an XRE motif (Figure 1) suggesting a plausible direct regulation of this promoter by AhR. Moreover, it has been described that TCDD activates NRF2 in target genes that contain XRE (xenobiotic responsive element) and/or ARE binding sequences.^42−44^

NF-kB, a key transcription factor, is involved in the dual regulation of *glast* and can also form a complex with AhR. As we previously reported,^30^ Aspartic acid has a negative effect on *chglast* transcription and as shown in panel C of Figure 6, it increases the NF-kB transcriptional activity of the reporter gene, supporting the idea of a TCDD-triggered negative role of NF-kB on *chglast* transcription. In a similar manner, the ARE sequences were activated upon the TCDD treatment. NF-kB regulates *chglast* promoter with stimulatory or repressive effects depending on the cellular context and the interaction with other transcription factors.

Interestingly, it has been reported that in AhR KO mice, a rapid loss of NF-kB occurs.^45^ The molecular AhR/NF-kB interaction found herein upon TCDD exposure is in line with the NF-kB decrease in these mice. Furthermore, a specific NF-kB inhibitor (caffeic acid) blocks the effects of TCDD-AhR activation and, therefore, the negative regulation on *chglast* mRNA levels (Figure 6D). These results suggest that the negative regulation of *chglast* transcription after a long-term TCDD exposure is mediated by AhR and NF-kB. The EMSA results shown in Figure 7 further support this notion, TCDD treatment clearly increases NF-κΒ nuclear extracts binding to their target sites of the *chglast* promoter. Interestingly, we were able to detect the binding complexes in BGC nuclear extract for the AhR target site (XRE sequence) from the *chglast* promoter and ARE sequence (NRF2 target site) from the AhR promoter (Figure 7, panels B and C). However. these complexes did not have significant changes by the TCDD treatment in BGC. A plausible scenario is that TCDD-activated AhR forms a dimer complex with NF-κΒ members and by these means regulates the *chglast* transcription as has been reported for various interleukin genes and the c-Myc promoter.^17,46^ Moreover TCDD treatment augments the binding to the AhR promoter ARE sites, presumably increasing AhR expression (Figure 7 panel C).

Taken together, in the present contribution, we demonstrate that an AhR agonist regulates Glu uptake in cerebellar BGC. Exposure to relevant concentrations of the dioxin TCDD results in a time and dose-dependent decrease in *chglast* mRNA levels, and in [^3^H]-d-Asp uptake as an index of GLAST/EAAT1 function. Since AhR is a sensor of a variety of neurotoxic pollutants, our results broaden our knowledge of the molecular mechanisms involved in the deleterious effect of these kinds of xenobiotics. Although the term *neurotoxic* refers to damage to neuronal cells, it is clear from our results, that glia cells are also vulnerable to these compounds. In fact, since the functionality of GLAST/EAAT1 is compromised, an extracellular Glu accumulation takes place, leading most possibly to an excitotoxic insult. Whether the glia-related excitotoxic insult is more severe damage to the neurons and oligodendrocytes than the well-documented reactive oxygen species generation upon dioxin exposure^47,48^ it is not known at this moment. In any event, we can propose that the dioxin-triggered granule cell death^21^ is, at least in part, glia-mediated, and therefore, we could define as well a *gliotoxic* effect.

## Methods

### Reagents

TCDD was purchased from AccuStandard (New Haven, CT, USA), [^3^H]-d-Asp (13 Ci/mmol) was from PerkinElmer (MA, USA), and MTT, d-aspartate, Actinomycin D (Act) Triton-x100, and dimethyl sulfoxide (DMSO) were purchased from Sigma-Aldrich. The Bradford reagent was obtained from Bio-Rad (Hercules, CA, USA), whereas caffeic acid was purchased from Abcam (Boston, MA, USA).

### *In Silico* Analysis

The promoter sequence of *chglast* previously reported^26^ GenBank Access: AY190600.1 and, the XRE sequence (5′-TNGCGTG-3′)^16^ were used on Genome.jp by Multiple sequences by CLUSTALW (https://www.genome.jp/tools-bin/clustalw).

### Cell Culture and Stimulation Protocol

Primary Bergmann glia cells (BGC) cultures were obtained after minor modifications from our previously described protocol.^49^ Cells were seeded (0.5 × 10^6^ cell/cm^2^) in Dulbecco’s modified Eagle’s medium (DMEM) supplemented with 10% fetal bovine serum (FBS), 2 mM glutamine, and 50 mg/mL gentamicin at 37 °C under standard conditions (5% CO_2_ and 95% humidity) and used on the fourth day after culture. Prior to any treatment, the cells were shifted to low serum (0.5% FCS) media for 2 h. TCDD was dissolved in dimethyl sulfoxide (DMSO) and diluted in a culture medium to the different concentrations used. It should be noted that special care was taken so that the DMSO concentration never exceeded 0.02%. In the case of the signaling analysis, inhibitors were added 30 min prior to the agonists. Experiments were carried out in triplicate and the reported results are from at least three independent experiments.

### Cell Viability Assay

Cell viability was measured with the 3-(4,5-dimethylthiazol-2-yl)-2,5-diphenyltetrazolium bromide assay. Control and BGC treated were incubated with 20 μL of 5 mg/mL MTT stock solution 3 h before the end of each condition and maintained at 37 °C. At the end of the treatment, the medium was discarded and 50 μL of dimethyl sulfoxide (DMSO) was added to each well to dissolve the formazan crystals. Absorbance was measured with an Infinite M200 PRO (TECAN) apparatus at 560 and 630 nm. Experiments were performed in quadruplicate in three independent cultures. 1% Triton X-100 was used as a positive control of cell death.

### [^3^H]-d-Aspartate Uptake

[^3^H]-d-Aspartate uptake was performed as previously described.^29^ BGC monolayers were seeded in 24-well dishes (0.5 × 10^6^ cell/cm^2^) and treated with different stimuli (indicated in each figure) for the indicated time periods. Once the incubation finished, BGC cultured monolayers were incubated at 37 °C with uptake buffer (HEPES-buffered solution containing 25 mM HEPES, 130 mM NaCl, 5.4 mM KCl, 1.8 mM CaCl_2_, 0.8 mM MgCl_2_, 33.3 mM glucose, and 1 mM NaH_2_PO_4_, pH 7.4), containing 0.4 μCi/mL [^3^H]-d-Asp (specific activity: 12.2 Ci/mmol, PerkinElmer, MA, USA). The d-Asp final concentration in the uptake was either 13 nM or 50 μM as annotated in each figure, as expected in the Michaelis–Menten analysis. The uptake assay was finished after 30 min of incubation, and monolayers were washed with ice-cold uptake buffer; cells were lysed with 250 μL of 0.1 N NaOH. Protein concentration in the lysates was determined with Bradford protein assay (Bio-Rad, CA, USA) and then transferred to scintillation vials. Radioactivity was measured in a PerkinElmer Tri-Carb 2810TR liquid scintillation counter (PerkinElmer, MA, USA). Experiments were performed in quadruplicates in three independent cultures.

### Transient Transfections and Luciferase assays

The reporter vectors to analyze the NF-kappaB and ARE activities were constructed by cloning a five-repeat consensus sequence (5′-GGGGAATTTCC-3′, and 5′-ATGACTCAGCA-3′, respectively) into the pGL3 Luciferase promoter vector described before.^30^ BGC were seeded in 24-well plates and transfected with 150 ng of pGL3Luc-5XNF-kappaB (pNF-kB Luc) or pGL3Luc-5XARE (pARE Luc) using Lipofectamine 3000 (Invitrogen). Briefly, the plasmids were mixed with Optimem, the p300 reagent, and Lipofectamine 3000. The mixture was incubated for 10 min at room temperature. Eighteen hours post-transfection, cells were further incubated for 24 h and treated with d-Asp or TCDD, as indicated in each figure. Luciferase assays were performed with a Luciferase Assay System (Promega). For protein lysates, cells were resuspended in 100 μL of reporter lysis buffer and lysed by two freeze–thawing cycles. Then, equal volumes of protein lysates were incubated with the Luciferase assay reagent. Detection was performed with an Infinite M200 PRO instrument (TECAN).

### Real-Time RT-PCR (RT-qPCR)

BGC were seeded in 24-well culture dishes and treated with d-Asp or TCDD for different time periods, as indicated in each figure. Total RNA was extracted with the Directzol RNA Miniprep Kit (Zymo Research). RT-qPCR was performed with the KAPA SYBR FAST One-Step RT-qPCR Kit (Kapa Biosystems) in a reaction volume of 10 μL (20 ng of total RNA, dNTP (10 nM), Rox reference dye (1×), Kapa RT mix (1×) Kappa SYBR master mix, forward primer (200 nM), and reverse primer (200 nM)). The reaction was performed in ABI Step One Plus Real-Time PCR System (Applied Biosystems) and consisted of a cDNA synthesis step at 42 °C for 5 min followed by inactivation of the RT at 95 °C for 5 min and then 40 cycles at 95 °C for 3 s and 60 °C for 30 s. Melting curves were constructed to determine the purity and to verify whether the bands corresponded with theoretical melting temperatures. To quantify mRNA levels, we used previously designed oligonucleotides:

*GLAST* Forward 5′- GGCTGCGGGCATTCCTC-3′

*GLAST* Reverse 5′-CGGAGACGATCCAAGAACCA-3′

As an endogenous control, we amplified the ribosomal protein *S17* mRNA with the following primers:

*S17* Forward 5′-CCGCTGGATGCGCTTCATCAG-3′

*S17* Reverse 5′-TACACCCGTCTGGGCAAC-3′

The relative quantification was performed by the comparative C_T_ (DDC_T_) method. Measurements were normalized using the endogenous control (S17 in this case); before the experiments were performed, specific primers to amplify chS17 and *chglast* genes were validated using a dynamic range and standard curves to determine the efficiency. The *chglast* primers showed a 90% efficiency (*R*^2^ = 0.99 and Slope = −2.55), and the chs17 primers showed a 90% of efficiency (*R*^2^ = 0.9997 and Slope = −3.24). Finally, in validating the efficiency between the target and endogenous gene, the dynamic range curve showed a slope of −0.02, which was validated using the DDCT method.

#### Electrophoretic Mobility Shift (EMSA) Assays

Nuclear extracts from 2.8 × 10^6^ BG cells were prepared as described previously.^24^ All buffers were freshly prepared, and the protease inhibitor phenylmethanesulfonyl fluoride (PMSF, 100 μg/mL, SIGMA Aldrich), and complete protease inhibitor cocktail (Roche) were added to prevent nuclear factor proteolysis. Protein concentration was measured by the Bradford method. The binding reactions were performed with nuclear extract (30 μg) from BGC (treated as indicated), and binding buffer (1 mM of DTT, 50 ng/mL of poly[deoxyinosinic-deoxycytidylic], 5 mM of MgCl_2_ (EMSA kit, Thermo Scientific); the reaction was incubated with 100 nM of Biotin-labeled double-stranded oligonucleotides (see Table 1) for 20 min and electrophoresed through 6.5% polyacrylamide gels using a low-ionic strength 0.5× Tris/Borate/EDTA buffer. For competition assays, each reaction was preincubated with the nonlabeled oligonucleotide (10×, cold probe) for 15 min before adding labeled DNA.

The gels were electrophoretically transferred to a nitrocellulose membrane after the membranes were cross-linked for 15 min with the E-gel transilluminator (Invitrogen). The DNA/protein complexes were detected by chemiluminescent nucleic acid detection module as indicated in the protocol (Thermo Scientific). Images were documented with a Fusion FX-Vilber Lourmat.

## Abbreviations

AMPA: α-amino-3-hydroxy-5-methyl-4-isoxazolepropoionic acid
AhR: aryl hydrocarbon receptor CNS
BGC: Bergmann glial cells, central nervous system
EMSA: electrophoretic mobility shift assay
EAATs: excitatory amino acid transporters
Glu: glutamate
GRI: ionotropic Glu receptors
GRM: metabotropic Glu receptors
GLAST: sodium-dependent Glu/aspartate transporter
GLT-1: Glu transporter 1
NMDA: *N*-methyl-d-aspartate
KA: Kainate
PAS: Per-Arnt-Sim
XRE: xenobiotic response element
TCDD: 2,3,7,8-tetracloro-p-dioxin.

## References

[ref1] Hansen K. B.; Wollmuth L. P.; Bowie D.; et al. Structure, Function, and Pharmacology of Glutamate Receptor Ion Channels. Pharmacol. Rev. 2021, 73 (4), 298–487. 10.1124/pharmrev.120.000131. 34753794 PMC8626789

[ref2] Haduch A.; Bromek E.; Puklo R.; Jastrzebska J.; Danek P. J.; Daniel W. A. The Effect of the Selective N-methyl-D-aspartate (NMDA) Receptor GluN2B Subunit Antagonist CP-101,606 on Cytochrome P450 2D (CYP2D) Expression and Activity in the Rat Liver and Brain. Int. J. Mol. Sci. 2022, 23 (22), 13746 10.3390/ijms232213746. 36430225 PMC9691159

[ref3] Sonnenberg J. L.; Mitchelmore C.; Macgregor-Leon P. F.; Hempstead J.; Morgan J. I.; Curran T. Glutamate receptor agonists increase the expression of Fos, Fra, and AP-1 DNA binding activity in the mammalian brain. J. Neurosci Res. 1989, 24 (1), 72–80. 10.1002/jnr.490240111. 2553994

[ref4] Martinez-Lozada Z.; Guillem A. M.; Robinson M. B. Transcriptional Regulation of Glutamate Transporters: From Extracellular Signals to Transcription Factors. Adv. Pharmacol. 2016, 76, 103–45. 10.1016/bs.apha.2016.01.004. 27288076 PMC5544923

[ref5] Lewerenz J.; Maher P. Chronic Glutamate Toxicity in Neurodegenerative Diseases-What is the Evidence?. Front Neurosci. 2015, 9, 469 10.3389/fnins.2015.00469. 26733784 PMC4679930

[ref6] Meldrum B.; Garthwaite J. Excitatory amino acid neurotoxicity and neurodegenerative disease. Trends Pharmacol. Sci. 1990, 11 (9), 379–87. 10.1016/0165-6147(90)90184-A. 2238094

[ref7] Rodriguez-Campuzano A. G.; Ortega A. Glutamate transporters: Critical components of glutamatergic transmission. Neuropharmacology. 2021, 192, 108602 10.1016/j.neuropharm.2021.108602. 33991564

[ref8] Zhou Y.; Danbolt N. C. GABA and Glutamate Transporters in Brain. Front Endocrinol (Lausanne). 2013, 4, 165 10.3389/fendo.2013.00165. 24273530 PMC3822327

[ref9] Martinez-Lozada Z.; Ortega A. Glutamatergic Transmission: A Matter of Three. Neural Plast. 2015, 2015, 787396 10.1155/2015/787396. 26345375 PMC4539489

[ref10] Martinez-Lozada Z.; Guillem A. M.; Flores-Mendez M.; et al. GLAST/EAAT1-induced glutamine release via SNAT3 in Bergmann glial cells: evidence of a functional and physical coupling. J. Neurochem. 2013, 125 (4), 545–54. 10.1111/jnc.12211. 23418736

[ref11] Pawlik M. J.; Obara-Michlewska M.; Popek M. P.; et al. Pretreatment with a glutamine synthetase inhibitor MSO delays the onset of initial seizures induced by pilocarpine in juvenile rats. Brain Res. 2021, 1753, 147253 10.1016/j.brainres.2020.147253. 33422530

[ref12] Danbolt N. C. Glutamate uptake. Prog. Neurobiol. 2001, 65 (1), 1–105. 10.1016/S0301-0082(00)00067-8. 11369436

[ref13] Martinez-Lozada Z.; Hewett S. J.; Zafra F.; Ortega A. Editorial: The known, the unknown, and the future of glutamate transporters. Front Cell Neurosci. 2022, 16, 1005834 10.3389/fncel.2022.1005834. 36060278 PMC9433117

[ref14] Taylor B. L.; Zhulin I. B. PAS domains: internal sensors of oxygen, redox potential, and light. Microbiol Mol. Biol. Rev. 1999, 63 (2), 479–506. 10.1128/MMBR.63.2.479-506.1999. 10357859 PMC98974

[ref15] McIntosh B. E.; Hogenesch J. B.; Bradfield C. A. Mammalian Per-Arnt-Sim proteins in environmental adaptation. Annu. Rev. Physiol. 2010, 72, 625–45. 10.1146/annurev-physiol-021909-135922. 20148691

[ref16] Rothhammer V.; Quintana F. J. The aryl hydrocarbon receptor: an environmental sensor integrating immune responses in health and disease. Nat. Rev. Immunol. 2019, 19 (3), 184–197. 10.1038/s41577-019-0125-8. 30718831

[ref17] Vogel C. F.; Matsumura F. A new cross-talk between the aryl hydrocarbon receptor and RelB, a member of the NF-kappaB family. Biochem. Pharmacol. 2009, 77 (4), 734–45. 10.1016/j.bcp.2008.09.036. 18955032 PMC2688397

[ref18] Xu C. X.; Krager S. L.; Liao D. F.; Tischkau S. A. Disruption of CLOCK-BMAL1 transcriptional activity is responsible for aryl hydrocarbon receptor-mediated regulation of Period1 gene. Toxicol. Sci. 2010, 115 (1), 98–108. 10.1093/toxsci/kfq022. 20106950 PMC2855348

[ref19] Giani Tagliabue S.; Faber S. C.; Motta S.; Denison M. S.; Bonati L. Modeling the binding of diverse ligands within the Ah receptor ligand binding domain. Sci. Rep. 2019, 9 (1), 10693 10.1038/s41598-019-47138-z. 31337850 PMC6650409

[ref20] Silva-Parra J.; Sandu C.; Felder-Schmittbuhl M. P.; Hernandez-Kelly L. C.; Ortega A. Aryl Hydrocarbon Receptor in Glia Cells: A Plausible Glutamatergic Neurotransmission Orchestrator. Neurotox Res. 2023, 41 (1), 103–117. 10.1007/s12640-022-00623-2. 36607593

[ref21] Kim S. Y.; Yang J. H. Neurotoxic effects of 2,3,7,8-tetrachlorodibenzo-p-dioxin in cerebellar granule cells. Exp Mol. Med. 2005, 37 (1), 58–64. 10.1038/emm.2005.8. 15761253

[ref22] Collins L. L.; Williamson M. A.; Thompson B. D.; Dever D. P.; Gasiewicz T. A.; Opanashuk L. A. 2,3,7,8-Tetracholorodibenzo-p-dioxin exposure disrupts granule neuron precursor maturation in the developing mouse cerebellum. Toxicol. Sci. 2008, 103 (1), 125–36. 10.1093/toxsci/kfn017. 18227101

[ref23] Mockenhaupt K.; Tyc K. M.; McQuiston A.; et al. Yin Yang 1 controls cerebellar astrocyte maturation. Glia. 2023, 71, 2437–2455. 10.1002/glia.24434. 37417428 PMC10529878

[ref24] Rosas S.; Vargas M. A.; Lopez-Bayghen E.; Ortega A. Glutamate-dependent transcriptional regulation of GLAST/EAAT1: a role for YY1. J. Neurochem. 2007, 101 (4), 1134–44. 10.1111/j.1471-4159.2007.04517.x. 17394550

[ref25] Unger T.; Lakowa N.; Bette S.; Engele J. Transcriptional regulation of the GLAST/EAAT-1 gene in rat and man. Cell Mol. Neurobiol. 2012, 32 (4), 539–47. 10.1007/s10571-011-9790-2. 22252783 PMC11498413

[ref26] Lopez-Bayghen E.; Espinoza-Rojo M.; Ortega A. Glutamate down-regulates GLAST expression through AMPA receptors in Bergmann glial cells. Brain Res. Mol. Brain Res. 2003, 115 (1), 1–9. 10.1016/S0169-328X(03)00136-0. 12824049

[ref27] Safe S. H. Comparative toxicology and mechanism of action of polychlorinated dibenzo-p-dioxins and dibenzofurans. Annu. Rev. Pharmacol. Toxicol. 1986, 26, 371–99. 10.1146/annurev.pa.26.040186.002103. 3013079

[ref28] Ema M.; Ohe N.; Suzuki M.; et al. Dioxin binding activities of polymorphic forms of mouse and human arylhydrocarbon receptors. J. Biol. Chem. 1994, 269 (44), 27337–43. 10.1016/S0021-9258(18)46990-6. 7961644

[ref29] Ruiz M.; Ortega A. Characterization of an Na(+)-dependent glutamate/aspartate transporter from cultured Bergmann glia. Neuroreport. 1995, 6 (15), 2041–4. 10.1097/00001756-199510010-00021. 8580436

[ref30] Hernandez-Melchor D.; Ramirez-Martinez L.; Cid L.; Palafox-Gomez C.; Lopez-Bayghen E.; Ortega A. EAAT1-dependent slc1a3 Transcriptional Control depends on the Substrate Translocation Process. ASN Neuro. 2022, 14, 17590914221116574 10.1177/17590914221116574. 35903937 PMC9340397

[ref31] Wong D.; Teixeira A.; Oikonomopoulos S.; et al. Extensive characterization of NF-kappaB binding uncovers non-canonical motifs and advances the interpretation of genetic functional traits. Genome Biol. 2011, 12 (7), R70 10.1186/gb-2011-12-7-r70. 21801342 PMC3218832

[ref32] Rushmore T. H.; Morton M. R.; Pickett C. B. The antioxidant responsive element. Activation by oxidative stress and identification of the DNA consensus sequence required for functional activity. J. Biol. Chem. 1991, 266 (18), 11632–9. 10.1016/S0021-9258(18)99004-6. 1646813

[ref33] Rothstein J. D.; Dykes-Hoberg M.; Pardo C. A.; et al. Knockout of glutamate transporters reveals a major role for astroglial transport in excitotoxicity and clearance of glutamate. Neuron. 1996, 16 (3), 675–86. 10.1016/S0896-6273(00)80086-0. 8785064

[ref34] Kim S. Y.; Choi S. Y.; Chao W.; Volsky D. J. Transcriptional regulation of human excitatory amino acid transporter 1 (EAAT1): cloning of the EAAT1 promoter and characterization of its basal and inducible activity in human astrocytes. J. Neurochem. 2003, 87 (6), 1485–98. 10.1046/j.1471-4159.2003.02128.x. 14713304

[ref35] Karki P.; Kim C.; Smith K.; Son D. S.; Aschner M.; Lee E. Transcriptional Regulation of the Astrocytic Excitatory Amino Acid Transporter 1 (EAAT1) via NF-kappaB and Yin Yang 1 (YY1). J. Biol. Chem. 2015, 290 (39), 23725–37. 10.1074/jbc.M115.649327. 26269591 PMC4583050

[ref36] Suzuki K.; Ikegaya Y.; Matsuura S.; Kanai Y.; Endou H.; Matsuki N. Transient upregulation of the glial glutamate transporter GLAST in response to fibroblast growth factor, insulin-like growth factor and epidermal growth factor in cultured astrocytes. J. Cell Sci. 2001, 114 (Pt 20), 3717–3725. 10.1242/jcs.114.20.3717. 11707523

[ref37] Lee E. S.; Sidoryk M.; Jiang H.; Yin Z.; Aschner M. Estrogen and tamoxifen reverse manganese-induced glutamate transporter impairment in astrocytes. J. Neurochem. 2009, 110 (2), 530–44. 10.1111/j.1471-4159.2009.06105.x. 19453300 PMC3920654

[ref38] Aguirre G.; Rosas S.; Lopez-Bayghen E.; Ortega A. Valproate-dependent transcriptional regulation of GLAST/EAAT1 expression: involvement of Ying-Yang 1. Neurochem. Int. 2008, 52 (7), 1322–31. 10.1016/j.neuint.2008.01.015. 18336953

[ref39] Erikson K.; Aschner M. Manganese causes differential regulation of glutamate transporter (GLAST) taurine transporter and metallothionein in cultured rat astrocytes. Neurotoxicology. 2002, 23 (4–5), 595–602. 10.1016/S0161-813X(02)00012-8. 12428731

[ref40] Walker M. K.; Heid S. E.; Smith S. M.; Swanson H. I. Molecular characterization and developmental expression of the aryl hydrocarbon receptor from the chick embryo. Comp Biochem Physiol C Toxicol Pharmacol. 2000, 126 (3), 305–19. 10.1016/S0742-8413(00)00119-5. 11048681

[ref41] Yasui T.; Kim E. Y.; Iwata H.; et al. Functional characterization and evolutionary history of two aryl hydrocarbon receptor isoforms (AhR1 and AhR2) from avian species. Toxicol. Sci. 2007, 99 (1), 101–17. 10.1093/toxsci/kfm139. 17556759

[ref42] Park E. Y.; Rho H. M. The transcriptional activation of the human copper/zinc superoxide dismutase gene by 2,3,7,8-tetrachlorodibenzo-p-dioxin through two different regulator sites, the antioxidant responsive element and xenobiotic responsive element. Mol. Cell. Biochem. 2002, 240 (1–2), 47–55. 10.1023/A:1020600509965. 12487371

[ref43] Radjendirane V.; Jaiswal A. K. Antioxidant response element-mediated 2,3,7,8-tetrachlorodibenzo-p-dioxin (TCDD) induction of human NAD(P)H:quinone oxidoreductase 1 gene expression. Biochem. Pharmacol. 1999, 58 (10), 1649–55. 10.1016/S0006-2952(99)00245-2. 10535757

[ref44] Nault R.; Doskey C. M.; Fader K. A.; Rockwell C. E.; Zacharewski T. Comparison of Hepatic NRF2 and Aryl Hydrocarbon Receptor Binding in 2,3,7,8-Tetrachlorodibenzo-p-dioxin-Treated Mice Demonstrates NRF2-Independent PKM2 Induction. Mol. Pharmacol. 2018, 94 (2), 876–884. 10.1124/mol.118.112144. 29752288 PMC6022803

[ref45] Thatcher T. H.; Maggirwar S. B.; Baglole C. J.; et al. Aryl hydrocarbon receptor-deficient mice develop heightened inflammatory responses to cigarette smoke and endotoxin associated with rapid loss of the nuclear factor-kappaB component RelB. Am. J. Pathol. 2007, 170 (3), 855–64. 10.2353/ajpath.2007.060391. 17322371 PMC1864867

[ref46] Kim D. W.; Gazourian L.; Quadri S. A.; Romieu-Mourez R.; Sherr D. H.; Sonenshein G. E. The RelA NF-kappaB subunit and the aryl hydrocarbon receptor (AhR) cooperate to transactivate the c-myc promoter in mammary cells. Oncogene 2000, 19 (48), 5498–5506. 10.1038/sj.onc.1203945. 11114727

[ref47] Sas K.; Szabó E.; Vécsei L. Mitochondria, Oxidative Stress and the Kynurenine System, with a Focus on Ageing and Neuroprotection. Molecules 2018, 23, 191 10.3390/molecules23010191. 29342113 PMC6017505

[ref48] Reichard J. F.; Dalton T. P.; Shertzer H. G.; Puga A. Induction of oxidative stress responses by dioxin and other ligands of the aryl hydrocarbon receptor. Dose Response 2006, 3 (3), 306–331. 10.2203/dose-response.003.03.003. 18648615 PMC2475945

[ref49] Ortega A.; Eshhar N.; Teichberg V. I. Properties of kainate receptor/channels on cultured Bergmann glia. Neuroscience. 1991, 41 (2–3), 335–49. 10.1016/0306-4522(91)90331-H. 1714547

